# World’s Largest Tonsillolith Removal: A Case Report With Literature Review of Large Tonsil Stones on Record

**DOI:** 10.1155/crot/5530230

**Published:** 2026-02-16

**Authors:** Anukaran Mahajan, Anubhuti Dhanuka, Karunesh Gupta

**Affiliations:** ^1^ ENT Department, Chikitsa ENT Hospital, Amritsar, 143001, India

## Abstract

Tonsil stones or tonsilloliths are mineralised concretions in the crypts of palatine tonsils. They are usually small and asymptomatic. Sometimes, they can be recurrent, large in size and produce symptoms like a sense of foreign body in throat, mild pain and heaviness in throat and halitosis. In such cases, a surgical intervention may be required for permanent cure. We came across one such patient with massive tonsillolith which mimicked a peritonsillar abscess initially. Eventually, the large tonsillolith was surgically removed in toto. It measured 5.2 × 2.5 × 2.5 cm which makes it the largest tonsillar stone ever to be successfully removed till date.

## 1. Introduction

Tonsil stones or tonsilloliths are calcifications of the debris in the tonsils, almost exclusively affecting palatine tonsils. Palatine tonsils have crypts and clefts which often entrap food particles, dead cells and bacteria. Over time, this debris can get mineralised into whitish concretions and is called as tonsil stones or tonsilloliths.

Tonsilloliths are usually small and recurrent and can be unilateral or bilateral. These are often asymptomatic. Symptoms, if present, can be one or more of the following:

- Throat irritation/discomfort.
- Foreign body sensation in throat.
- Mild pain or heaviness in throat with mild difficulty in swallowing food.
- Halitosis.

Tonsilloliths usually do not require any treatment except routine warm saline gargles and maintaining oral hygiene. In some cases, surgery in the form of tonsillectomy and/or enucleation of tonsil stone may be required where symptoms are persistent and troublesome to the patient.

As mentioned earlier, tonsil stones are small in size (less than 0.5 cm). Rarely, these tonsil stones may present as large masses of size > 1 cm. Various authors, all over the world, have reported tonsilloliths of sizes ranging from 1 to 4 cm [1–44].

We also present a case of a large unilateral tonsillolith of size 5.2 × 2.5 × 2.5 cm which was surgically removed. On reviewing the literature, we found it to be the largest tonsil stone ever to be surgically removed till date.

## 2. Case Report

**FIGURE 1:**
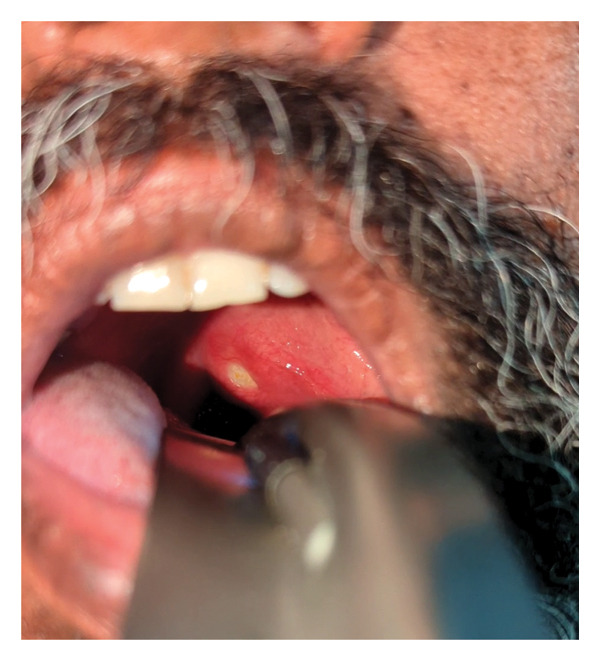
Bulge in the left peritonsillar region with an apparent ‘pus point’. In reality, the tonsillolith is mimicking a peritonsillar abscess.

**FIGURE 2:**
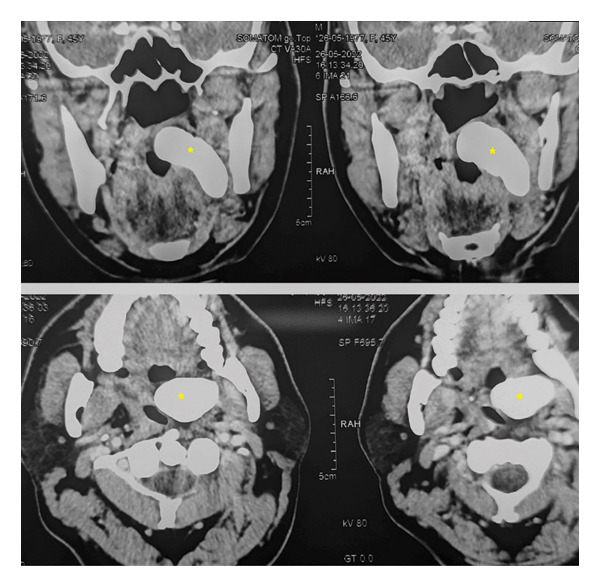
CT scan showing a large tonsillolith.

**FIGURE 3:**
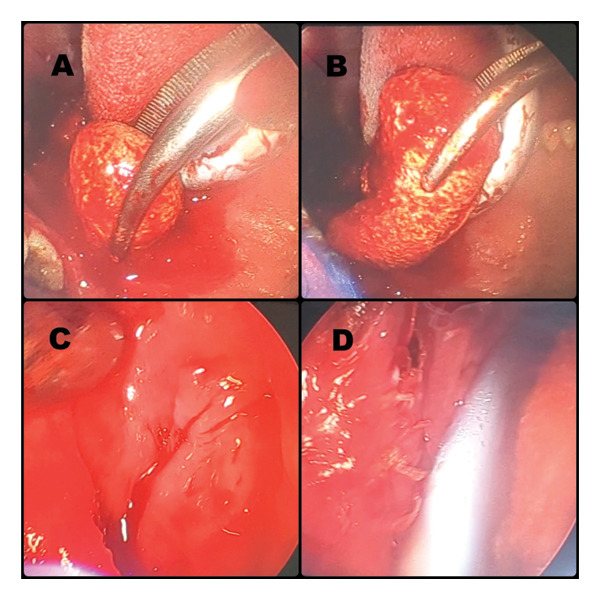
Intraoperative images. Tonsillolith being delivered out (A, B). Healthy underlying cavity where tonsillolith was situated (C). Sutured peritonsillar incision using absorbable sutures (D).

**FIGURE 4:**
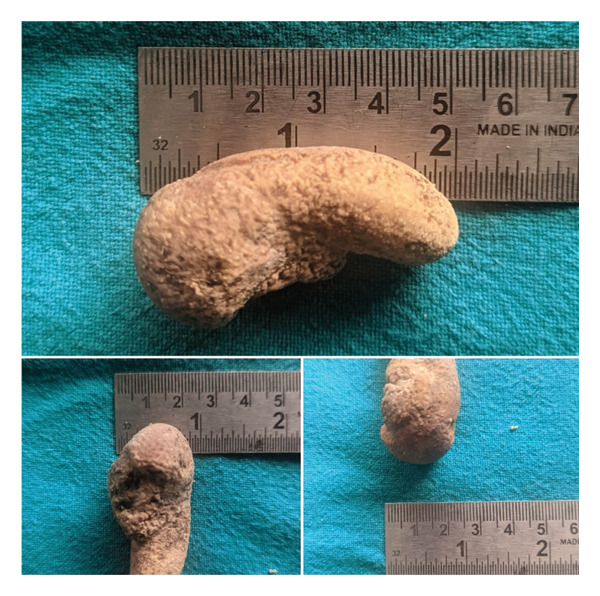
Excised tonsillolith specimen.

We present a case of a 48‐year‐old male patient who presented with complaints of mild pain and heaviness in the left side of throat. It was accompanied by mild dysphagia and some foreign body sensation. On examining the oral cavity, a bulge was seen in the left peritonsillar region. A preliminary diagnosis of peritonsillitis was made, and the patient was prescribed a short course of antibiotics and follow‐up. The patient did not show up on his scheduled follow‐up date, only to revisit after 4  months with persisting complaints of mild pain, heaviness and dysphagia. Oral cavity examination again revealed a left peritonsillar bulge, but this time, it was accompanied by an apparent pus point over it (Figure 1). Although there were no constitutional symptoms, a working diagnosis of peritonsillar abscess was made and antibiotics were started. Transoral aspiration was attempted, but the needle could not penetrate into the so thought abscess pocket and was instead encountering a stony‐hard lesion. Computed tomography (CT) scan was advised to further ascertain the pathology. To our surprise, CT revealed a large calcified mass lesion in the peritonsillar region (Figure 2). A final diagnosis of a large tonsillolith was made. Retrospectively, it fitted all the symptoms and examination findings. After counselling the patient, he was taken into the operating room. Tonsillolith was successfully removed in toto through transoral route using a peritonsillar incision (Figures 3 and 4). The removed specimen was sent for histopathology. The healthy tonsil was left undisturbed as it was the first episode, and the authors considered it prudent to give the tonsils a chance to heal in the most physiological manner. The incision site was sutured with absorbable sutures, and oral diet was started the next day, much to the symptomatic relief of the patient. Histopathology revealed bacterial colonies with entrapped neutrophils and dystrophic calcification (Figures 5(a) and 5(b)). A microbial culture was not performed during histopathological examination. The patient is under our regular follow‐up and disease free for 1 year now.

FIGURE FIGURE​ 5Histopathology of the removed tonsillolith specimen. (a) Histopathology of calcification (marked with green arrow). (b) Histopathology of bacterial colonies (green arrow) and neutrophils (orange arrow).

**Figure:**
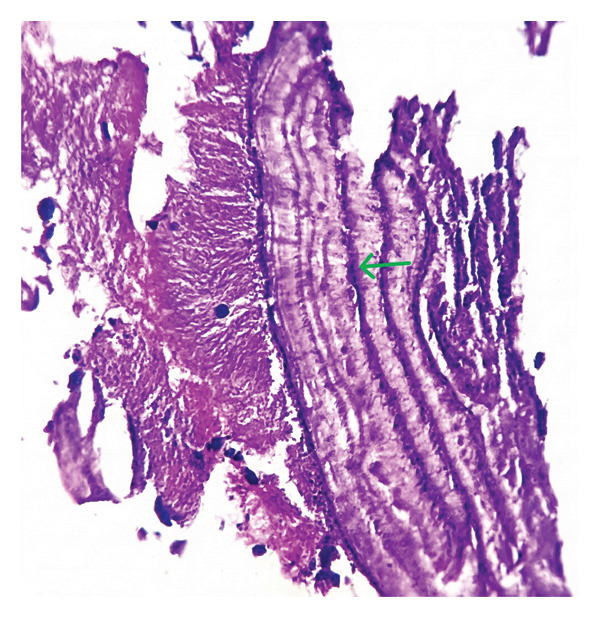
(a)

**Figure:**
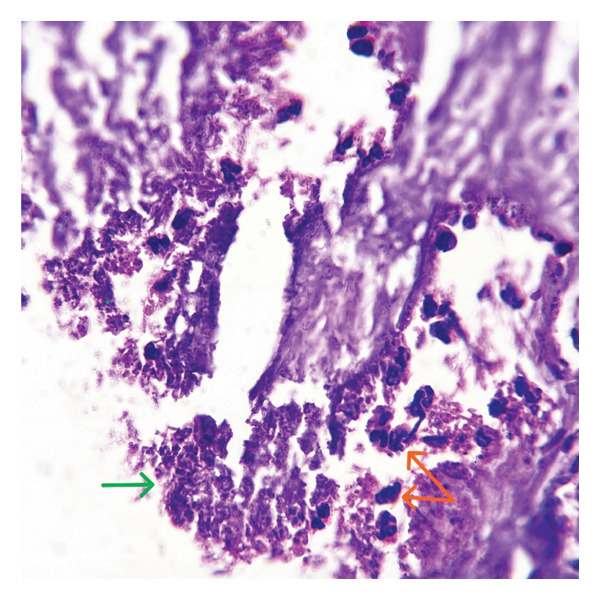
(b)

## 3. Discussion

Tonsil stones or tonsilloliths are mineralised concretions in the crypts of palatine tonsils. Small tonsilloliths usually do not require any surgical intervention. Warm saline gargles, especially done post‐meals, are enough to wash away small stones. For larger tonsilloliths, or, when the symptoms are persistent, a surgical option can alleviate the disease [8–10, 19, 23, 26, 28, 29, 31, 33, 34, 37–43]. Our patient had an enormous tonsillolith which needed a transoral enucleation procedure.

Tonsilloliths are usually asymptomatic and may be diagnosed as an incidental finding [18, 23, 25, 33, 35, 36]. Rarely, they can present with one or more of the following symptoms: foreign body sensation in throat, mild pain, mild dysphagia, throat heaviness, sore throat and halitosis [1–14, 17, 19–22, 24, 26, 27, 30, 31, 34, 37–43]. Most of these symptoms are recurrent. Our patient presented with similar complaints of mild throat pain and heaviness, mild dysphagia and foreign body sensation. Multiple symptoms could be owing to the large size of the tonsillolith (5.2 × 2.5 × 2.5 cm).

Over the years, various authors have described large tonsil stones. We did an extensive review of literature and have enumerated the large tonsilloliths in Table 1.

**TABLE 1:** Large tonsil stones described in the literature (in chronological order) (M = male; F = female; NA = not available; NR: not reported).

Author	Year	Age/sex	Location	Weight	Size	Signs and symptoms	Treatment
Swain [1]	1920	56/F	Right	NA	Large	Pain, dysphagia, enlarged tender lymph nodes, recurrent stones	NR
Woodman [2]	1920/21	64/M	Right	6.2 g	NA	‘Stone in the mouth’, abscess, neck sinus, recurrent stones	NR
Goodman [3]	1930	20/M	Left	3.3 g	2.1 ∗ 1.5 ∗ 1.54 cm	Sore throat	NR
Rubin [4]	1936	32/M	Right	24.7 g	3.7 ∗ 2.8 cm	Pain, lump in throat, TB	NR
Bugge [5]	1949	60/M	Bilateral	3.5 g	NA	Recurrent tonsillitis	NR
		45/M	Bilateral	4.5 g	NA	Sore throat	NR
Clarke [6]	1954	35/M	Right	13.3 g	NA	Neck sinus, lump in throat, pain, halitosis, pneumonia	NR
Harding [7]	1962	57/F	Right	NA	1 ∗ 1 cm, 0.5 cm	Sore throat	NR
Mishenkin [8]	1965	53/F	Palatine	2.7 g	3.4 ∗ 0.1 cm	Recurrent tonsillitis, pharyngeal heaviness	NR
		17/F	Palatine	3.3 g	2.5 ∗ 0.1 cm	Recurrent tonsillitis	NR
		33/M	Palatine	37 g	2.3 ∗ 2 cm	Pharyngeal heaviness	Prior tonsillectomy (at age 30)
Hiranandani [9]	1967	65/F	Right	42 g	2.5 ∗ 3 cm	Recurrent throat pain	Right tonsillectomy
Shrimali and Bhatia [10]	1972	63/F	Left	32 g	2.5 ∗ 2 cm	Throat pain, odynophagia	Enucleation under GA
Dale and Wing [11]	1974	54/F	Right	0.56 g	2 ∗ 1 cm	Halitosis	NR
Ramanjaneyulu [12]	1974	64/M	Right	NA	Large	Throat pain, cervical lymphadenopathy	Patient refused surgery
Samant and Gupta [13]	1975	45/M	Right	30 g	NA	Painless slow growing swelling, history of right peritonsillar abscess	
		16/M	Right	25 g	NA	Slow growing throat swelling, history of quinsy	
Gapany‐Gapanavicius [14]	1976	26/M	Right	6.7 g	3.2 ∗ 2.1 ∗ 1.7 cm	Recurrent sore throat, swelling, fever	NR
Hoffman [15]	1978	NA	Right	NA	NA	Intermittent swelling	NR
Kulinich [16]	1979	50/F	Right	NA	NA	Enlarged submandibular glands	NR
Elidan et al. [17]	1980	25/F	Right	5.2 g	2.5 ∗ 1.8 ∗ 1.5 cm	Recurrent throat infection, pain radiating to right ear	NR
Marshall and Irwin [18]	1981	NA	Left	NA	NA	Incidental finding	NR
Cooper et al. [19]	1983	77/F	Left	8 g	4 ∗ 2 ∗ 2 cm	Chronic oral infection, multiple episodes of pneumonia, right tonsillectomy and recurrent intermittent infection of left submandibular gland with abscess formation and Wharton’s duct calculi	Enucleation (prior tonsillectomy)
Padmanathan et al. [20]	1984	40/M	Left	Large stone: 22.6 g, small stone: 300 mg	4 ∗ 3 cm	History of recurrent swelling in the left submandibular region, throat pain	
Gadgil [21]	1984	28/M	Right	NA	NA	Throat pain, dysphagia and recurrent pharyngitis	NR
Hadi and Samara [22]	1985	28/M	Left	8 g	2 ∗ 1.5 ∗ 1 cm	Odynophagia and foreign body sensation in the throat	
Westmore and Hupp [23]	1988	63/M	Left	NA	1.5 ∗ 1.5 ∗ 3 cm	Emphysema and general ill health; died of pulmonary embolus 2 days after examination	Enucleation
Heppt [24]	1989	77/M	Right	7 g	NA	Mass in right tonsillar fossa	NR
Cerny and Bekarak [25]	1990	10/F	Tonsillar fossa	0.84 g	2.6 ∗ 0.4 cm	Asymptomatic	NR
Kimura et al. [26]	1993	27/M	Left	8.5 g	3 ∗ 2.6 ∗ 1.6 cm	Recurrent pharyngitis, tonsillitis, tonsillar abscess	Spontaneous expulsion and tonsillectomy
Vera Llao et al. [27]	1995	43/F	Right	NA	NA	Odynophagia	
Castellano and Marcolli [28]	1996	68/F	Left	14 g	2.8 ∗ 2.3 ∗ 2.1 cm	Lymphadenopathy left mandible	Enucleation under LA
Jones [29]	1996	70/M	Right	NA	1‐2 mm	Mass in right tonsillar fossa	Enucleation
el‐Sherif and Shembesh [30]	1997	24/M	Right	8.2 g	3.2 ∗ 2 ∗ 1.5 cm	History of recurrent throat infections (4 years duration) and throat pain	
Revel et al. [31]	1998	68/M	Right	NA	NA	Odynophagia and right otalgia	Right tonsillectomy
Modrzynski et al. [32]	2001	70/M	Right	NA	4.1 ∗ 2.1 ∗ 1.9 cm	Pharyngitis and recurrent tonsillitis, recurrent throat infection	
Neshat et al. [33]	2001	69/M	Left	NA	1.2 ∗ 1.6 cm	Asymptomatic	Enucleation under LA
Cogolludo Perez et al. [34]	2002	69/F	Right	NA	3 ∗ 2 ∗ 2.3 cm	Dysphagia	Tonsillectomy
Sezer et al. [35]	2003	31/F	Left	NA	1.5 ∗ 1.5 ∗ 1.3 cm	Right pericoronitis with pain and swelling; incidental X‐ray finding	
Ram et al. [36]	2003	57/F	Right	NA	NA	Asymptomatic; incidental X‐ray finding; squamous cell carcinoma of right buccal mucosa with pain	
Silvestre‐Donat et al. [37]	2005	55/F	Right	NA	2.5 ∗ 1.5 cm	Dysphagia	Enucleation
Thakur et al. [38]	2008	12/F	Left	NA	4.2 ∗ 3.6 ∗ 2.1 cm	Odynophagia, recurrent sore throat	Enucleation with tonsillectomy under GA
Gangaraj and Maruthi [39]	2013	56/F	Right	NA	2 ∗ 1.5 cm	Foreign body sensation in throat, halitosis	Enucleation
Neha Salaria et al. [40]	2016	35/F	Right	NA	2.6 ∗ 2.5 ∗ 2.3 cm	Recurrent episodes of tonsillitis since 1 year. There was associated difficulty in swallowing, along with a history of halitosis	Enucleation
Alfayez A et al. [41]	2018	45/M	Left	NA	3.1 ∗ 2.3 cm	Recurrent sore throat and tonsillitis	Enucleation with tonsillectomy
Khetani et al. [42]	2020	24/F	Left	NA	2 ∗ 1.17 cm	Foreign body sensation in throat, ear pain	Enucleation
Vijayan et al. [43]	2020	7/M	Bilateral	NA	1 ∗ 2 ∗ 2.3 cm on right Small on left	Sore throat, odynophagia and foreign body sensation in throat, recurrent tonsillitis	Tonsillectomy

*Note:* Adapted from Ram et al. [36], with additional cases from the current literature review.

It was evident from the review of literature that this was the largest tonsillolith to be ever removed successfully till date.

A few articles have (incorrectly) mentioned a tonsillolith of size 14.5 cm by Rubin in 1936 [44]. But the original publication by Rubin mentions a tonsillolith of size 1.5 inch × 1 inch only [4]. It was clear that the size was mistakenly cited by subsequent articles.

## Funding

No external funding was received for this article.

## Consent

Written informed consent was obtained from the patient for publication of this case report and accompanying images.

## Conflicts of Interest

The authors declare no conflicts of interest.

## Data Availability

The data that support the findings of this study are available on request from the corresponding author. The data are not publicly available due to privacy or ethical restrictions.
